# An ADAM10 promoter polymorphism is a functional variant in severe sepsis patients and confers susceptibility to the development of sepsis

**DOI:** 10.1186/s13054-015-0796-x

**Published:** 2015-03-05

**Authors:** Lili Cui, Yan Gao, Yuliu Xie, Yan Wang, Yujie Cai, Xin Shao, Xiaotang Ma, You LI, Guoda Ma, Gen Liu, Wanwen Cheng, Yu Liu, Tingting Liu, Qunwen Pan, Hua Tao, Zhou Liu, Bin Zhao, Yiming Shao, Keshen Li

**Affiliations:** Guangdong Key Laboratory of Age-Related Cardiac and Cerebral Diseases, Affiliated Hospital of Guangdong Medical College, Renmin street south 57, Xiashan district, Zhanjiang City, 524001 Guangdong Province PR China; The Intensive Care Unit, the Forth Affiliated Hospital of Harbin Medical University, Harbin, China; The Intensive Care Unit, Affiliated Hospital of Guangdong Medical College, Zhanjiang, PR China; Clinical Research Center of Guangdong Medical College, Affiliated Hospital of Guangdong Medical College, Zhanjiang, PR China

## Abstract

**Introduction:**

Although genetic variants of the A disintegrin and metalloproteinase 10 (ADAM10) gene have been shown to be associated with susceptibility to several inflammatory-related diseases, to date little is known about the clinical relationship in the development of sepsis.

**Methods:**

Two genetic variants in the promoter of ADAM10 were selected to analyze the potential association with the risk of sepsis. A total of 440 sepsis patients and 450 matched healthy individuals in two independent Chinese Han population were enrolled. Pyrosequencing and polymerase chain reaction-length polymorphism was used to determine the genotypes of the rs514049 and rs653765. A real-time qPCR method was used to detect the mRNA level of ADAM10. Enzyme-linked immunosorbent assay was used to measure the expression levels of substrates CX3CL1, interleukin (IL)-6R, tumor necrosis factor alpha (TNF-α), and the pro-inflammatory cytokines IL-1β and IL-6. Luciferase assay was used to analyze the activities of the promoter haplotypes of ADAM10.

**Results:**

No statistically significant differences between sepsis cases and controls in the genotype or allele frequencies were observed, suggesting that ADAM10 single nucleotide polymorphisms (SNPs) may not be risk factors for the occurrence of sepsis. A significant difference in the genotype and allele frequencies of the rs653765 SNP between patients with sepsis subtype and severe sepsis (*P* = 0.0014) or severe sepsis/sepsis shock (*P* = 0.0037) were observed. Moreover, the rs653765 CC genotype in severe sepsis showed a higher ADAM10 level compared to healthy groups, and the rs653765 CC polymorphism had a strong impact on the production of the ADAM10 substrates CX3CL1, IL-6R and TNF-α. Furthermore, the functional assay showed that ADAM10 C-A haplotype carriers exhibited significantly higher reporter activity compared with the T-A carriers and T-C carriers in human acute monocytic leukemia cell line.

**Conclusions:**

Our data initially indicated the ADAM10 rs653765 polymorphism was associated with the development of severe sepsis; the risk CC genotype could functionally affect the expression level of ADAM10 mRNA and was accompanied by the up-regulation of its substrates. Thus, ADAM10 might be clinically important and play a critical role in the pathogenesis of the development of sepsis, with potentially important therapeutic implications.

**Electronic supplementary material:**

The online version of this article (doi:10.1186/s13054-015-0796-x) contains supplementary material, which is available to authorized users.

## Introduction

Despite recent advances in antibiotic therapy and aggressive operative intervention, sepsis remains a difficult challenge in the ICU that displays a high mortality rate of 30% to 50% among severe sepsis and septic shock patients [1-3]. Although the pathophysiological mechanisms underlying sepsis are unclear, pro-inflammatory cytokines such as IL-6 and IL-1β, which are involved in the immune response, are clearly closely linked to the pathogenesis of sepsis [4,5]. Currently, vast evidence indicates that certain functional genetic variants, such as those in the genes of TNF-a, IL-6 and other inflammatory-related proteins, may be associated with the susceptibility to sepsis by influencing its inflammatory pathogenesis [6-9].

The A disintegrin and metalloproteinase (ADAM) proteins are type I membrane glycoproteins that contain 34 transmembrane domains, as well as a characteristic extracellular metalloproteinase domain and a disintegrin domain [10-12]. ADAM10, one member of the ADAM family, is involved in the shedding of dozens of substrates, such as CX3CL1, IL-6R, TNF-a and VE-cadherin, and this process plays a critical role in various inflammation-related diseases, including Alzheimer’s disease (AD), atherosclerotic cerebral infarction (ACI) and cancer [12-17]. To date, increasing evidence has demonstrated that ADAM10 affects the production of the inflammatory cytokines IL-6 and IL-1β in macrophages and thus, is implicated in the process of leukocyte migration and in the promotion of pro-inflammatory activity [18-20]. Some studies have revealed that ADAM10 inhibitors performed beneficial functions, such as increasing apoptosis resistance or suppressing the inflammatory immune response, in several cell models [21-23]. This evidence suggests that ADAM10 may play a vital role in the pathogenesis of directly inflammatory-related diseases, such as sepsis. The human ADAM10 gene is located on chromosome 15q21.3-q23 and contains 16 exons interrupted by 15 introns [24,25]. Several single-nucleotide polymorphisms (SNPs) are located in the promoter region of the ADAM10 gene, some of which are functional [26,27]. It has been reported that the ADAM10 rs514049-rs653765 C-A promoter polymorphisms decrease ADAM10 mRNA expression in the cerebrospinal fluid of patients with AD [28]. The ADAM10 rs653765 C > T polymorphism, located in the promoter region, also acts as a functional SNP that influences ADAM10 mRNA expression [29]. These SNPs are associated with the susceptibility to several diseases, such as ACI, AD, conduct disorder and others [29-31].

However, although several studies have suggested a correlation between ADAM10 and sepsis, to date, no study has examined the association between ADAM10 polymorphisms and sepsis. The present case-control study examined 440 sepsis patients and 450 healthy controls to determine whether the promoter variations of ADAM10 (rs653765 and rs514049) are associated with susceptibility to sepsis and whether these two functional SNPs influence ADAM10 expression in sepsis patients. Moreover, to investigate the effect of these ADMAM10 variants on the inflammation process, we analyzed the possible associations between these polymorphisms and the expression levels of downstream substrates of ADAM10 and pro-inflammatory cytokines in sepsis patients.

## Methods

### Study population

Our study consecutively enrolled 440 sepsis patients and 450 matched healthy controls from two regions of China between July 2009 and December 2013. This sample included 273 sepsis patients from the ICU and 280 healthy controls from the Health Examination Center of the Affiliated Hospital of Guangdong Medical College in southern China (Zhanjiang, China) and 167 sepsis patients and 170 healthy controls from Harbin Medical University in northern China (Harbin, China). The blood samples were collected upon the diagnosis of sepsis. The sepsis subtypes were defined according to the International Sepsis Definitions Conference in 2001 [32]. Patients with a history of malignant tumors, human immunodeficiency virus, autoimmune diseases, blood diseases, ACI or other organic diseases were excluded. All participants were members of the Chinese Han population and were older than 18 years of age. The participants in the healthy control group were free from any recent acute illness, any chronic illness such as autoimmune diseases, hypertension, diabetes, cancer or major cardiac, renal, hepatic, endocrinological disorders and any history of sepsis. No significant differences were observed in age or gender between the patients and the controls in the two districts (Table 1). Written informed consent was obtained from all of the enrolled participants, and this study was reviewed and approved by the Ethics Committee of the Affiliated Hospital of Guangdong Medical College (Zhanjiang, China) and the Fourth Affiliated Hospital of Harbin Medical University (Harbin, China).

**Table 1:** **Demographic characteristics of sepsis patients and healthy controls**

**Zhanjiang (Southern China)**	**Cases (n = 273)**	**Control (n = 280)**	***P***
Age, years, mean ± SD	62.9 ± 18.5	62.0 ± 14.7	0.661
Gender, male/female, number	200/73	213/67	0.447
**Harbin (Northern China)**	**Cases (n = 167)**	**Controls (n = 170)**	
Age, years, mean ± SD	58.4 ± 21.2	62.2 ± 11.4	0.551
Gender, male/female, number	90/77	85/85	0.475
**All subjects**	**Cases (n = 440)**	**Controls (n = 450)**	
Age, years, mean ± SD	61.3 ± 19.9	62.1 ± 13.5	0.631
Gender, male/female, number	290/150	298/152	0.921

### DNA isolation and genotyping

Genomic DNA was extracted from whole blood using the TIANamp Blood DNA Kit (TianGen Biotech, Beijing, China) according to the manufacturer’s instructions. The purified DNA was quantified in a spectrophotometer and was stored at −80°C until analysis. A SNaPshot Multiplex Kit was used to analyze the gene polymorphisms (Genesky Biotechnologies, Inc., Shanghai, China). Briefly, for rs653765-rs514049, the forward primer was AGCACCTCCCTCTCGCTCCAC, and the reverse primer was TGCATTTATGTTCGCATCACTGG. Polymerase chain reaction (PCR) was conducted in a final volume of 10 μL, which contained 5 μL of the SNaPshot Multiplex Kit reagent (ABI), 2 μL of the templates containing the multiplex PCR products, 1 μL of the primer mix and 2 μL of water. The PCR reaction protocol was as follows: after denaturation at 95°C for 2 minutes, 11 cycles of DNA amplification were performed using Taq PCR for 20 s at 94°C (denaturation), 40 s at 65°C (annealing), and 90 s at 72°C (extension), followed by 24 cycles of DNA amplification for 20 s at 94°C (denaturation), 30 s at 59°C (annealing), and 90 s at 72°C (extension). The amplified products were stored at 4°C. The extension products were purified for 1 h at 37°C via incubation in 1 U of shrimp alkaline phosphatase (Takara, Otsu, Shiga, Japan), followed by incubation for 15 minutes at 75°C to inactivate this enzyme. Additionally, 0.5 μL of the purified products was mixed with 9 μL of Hi-Di formamide and 0.5 μL of Liz120 Size Standard. Next, the samples were incubated for 5 minutes at 95°C and then inserted into the ABI Prism 3130XL genetic sequence analyzer. All individuals were successfully genotyped for the SNPs rs653765 and rs514049. In addition, 10% of the samples were randomly selected as the validation group for re-genotyping. The final data were analyzed using GeneMapper 4.1 (Applied Biosystems, Foster City, CA, USA).

### Mononuclear cell isolation and ELISA

We randomly selected 76 patients with sepsis and 84 healthy controls from the recruited individuals for the isolation of mononuclear cells. The 76 sepsis samples included 7 samples of sepsis, 49 samples of severe sepsis and 20 samples of septic shock. The genotype distribution of the rs653765 polymorphism in the severe sepsis patients was as follows: 36 patients carrying the CC genotype and 13 patients carrying the CT/TT genotype. The genotype distribution of rs514049 polymorphism was 44 patients carrying the AA genotype and 5 patients carrying the AC/CC genotype. Moreover, 64 and 22 healthy controls carried the CC and CT/TT genotypes of rs653765, respectively, and 74 and 10 healthy controls carried the AA and AC/CC genotypes of rs653765, respectively; these healthy controls were selected as the control group. Peripheral blood mononuclear cells (PBMCs) were isolated from 10 mL of peripheral blood by the density gradient centrifugation method using Lymphoprep™ (Axis-Shield PoCAS, Oslo, Norway). In short, an equal volume of 0.9% NaCl was added to the blood samples, and the diluted blood was layered above a Ficoll-Paque PREMIUM solution. Then, the samples were centrifuged at 800 × g for 30 minutes at room temperature, which caused the mononuclear cells to form a distinct band at the media interface. Subsequently, we transferred the cells to fresh tubes using Pasteur pipettes without removing the upper layer and then washed these cells with 0.9% NaCl. Finally, we centrifuged the samples again at 250 × g for 10 minutes, and the mononuclear cells that we obtained were stored at −80°C. ELISA was performed according to the manufacturer’s instructions to measure the levels of substrates and inflammatory cytokines in the supernatants of the isolated PBMCs. CX3CL1, TNF-α, IL-6R, IL-6 and IL-1β were measured in the PBMCs using each specific ELISA kit (R&D Systems, Minneapolis, MN, USA). Then, the absorbance of each sample was determined at 450 nm using a microplate reader, and the final cytokine levels were analyzed according to a standard curve. The minimum detectable levels of IL-1β, TNF-α, IL-6, IL-6R and CX3CL1 were 5 pg/mL, 8 pg/mL, 2 ng/L, 15 ng/L and 0.3 ng/mL, respectively.

### RNA extraction and real-time PCR

Total cellular RNA was extracted as soon as possible after the isolation of the mononuclear cells using the RNAprep Pure Blood Kit (Sangon Biotech, Shanghai, China) according to the manufacturer’s instructions. The RNA samples were assessed via agarose gel electrophoresis and were stored at −80°C. Then, equal amounts (5 μL) of total RNA were reverse-transcribed using a First Strand cDNA Synthesis Kit (Thermo Fisher Scientific, Waltham, MA, USA) according to the manufacturer’s instructions. The mRNA expression level of ADAM10 was determined via real time (RT)-PCR using the SYBR green method. The primers used in this assay were as follows: ADAM10 sense primer, CTGGCCAACCTATTTGTGGAA, and antisense primer, GACCTTGACTTGGACTGCACTG; GAPDH sense primer, GAAGGGCTCATGACCACAGTCCAT, and antisense primer, TCATTGTCGTACCAGGAAATGAGCTT. All measurements of ADAM10 were performed in triplicate. Briefly, the ADAM10-specific primers were diluted to a final concentration of 1 μM, and the final volume of 10 μL of the PCR amplification reaction was mixed with 5 μL of 2 × SYBR Green PCR master mix (TaKaRa), 0.2 μL of each specific forward and reverse primer, 3.6 μL of DNase-free water and 1 μL of cDNA as a template. The relative level of each transcript normalized to the housekeeping gene glyceraldehyde-3-phosphate dehydrogenase was determined. RT-PCR was performed using a LightCycler 480 sequence detector system (Roche Applied Science, Laval, Quebec, Canada) as follows: initial step of 95°C for 30 s, followed by 40 cycles of 95°C for 5 s and 60°C for 20 s. The relative expression levels of ADAM10 mRNA in all participants were obtained using the 2^-△△Ct^ method.

### Plasmid constructs

Based on our haplotype assay results, three haplotype variants of the ADAM10 3′UTR (rs514049-rs653765-A-C, rs514049-rs653765-A-T and rs514049-rs653765-C-T) were amplified from the promoter of the ADAM10 gene using PCR. Briefly, the AMA10 promoter gene fragments were amplified by PCR. The sequences of the primers were as follows: ADAM10: 5′- CTCGCAGTCGTGCCTCAC and 3′ -AATTCGGCCTACTCAAGCAC. The three ADAM10 promoter haplotypes in our study were amplified by PCR from the genomic DNA based on the known alleles of rs514049 and rs653765. Each amplified product was verified by dissociation curves and gel electrophoresis and was then cloned into the expression region of the pGL4.10 vector (Promega, Madison, Wisconsin, USA). After transformation into *Escherichium coli* DH5α cells, all of the plasmids were isolated and purified using a Plasmid Midi Kit (Promega, USA). The inserted fragments were confirmed by sequencing.

### Cell culture

The human acute monocytic leukemia cell line THP-1 (Shanghai Institute of Cell Biology, China) was cultured as cell suspensions in RPMI 1640 medium (HyClone, Logan, UT, USA) supplemented with 10% heat-inactivated FBS (Hyclone, USA), 100 U/ml penicillin, and 100 μg/mL streptomycin (Gibco-BRL, Life Technologies, Grand Island, NY, USA). The THP-1 cells were maintained at 37°C in a humidified incubator containing 5% CO_2_ during growth and treatment. The cells were subcultured at a split ratio from 1:2 to 1:3 and were passaged every 2 to 3 days. The treatments were administered to a density of 2 × 105 cells/mL in 12-well plates.

### Luciferase assay

THP-1 cells were transiently transfected for 48 h with the firefly luciferase pGL4.10 (luc2) haplotype reporter and Renilla luciferase pGL4.75 vectors using Lipofectamine 2000 (Invitrogen, USA) according to the manufacturer instructions. Three parallel samples were used in all transfections, and all experiments were performed in triplicate. The assays were performed according to the protocol of the dual-luciferase assay kit (Promega, USA). The luminescence was measured using a Mithras LB940 Multilabel Reader (Berthold Technologies, Bad Wildbad, Germany). The activity of Renilla luciferase was normalized to that of firefly luciferase.

### Statistical analyses

All data were analyzed using SPSS 17.0 and GraphPad Prism 4.0 (GraphPad Software, Inc., San Diego, CA, USA). The Benjamin-Hochberg procedure for multiple-testing correction was used to analyze the false discovery rate. The genotype and allele frequencies were calculated using the chi squared or Fisher’s exact test as appropriate. The distributions of the genotype frequencies for the two SNPs were consistent with Hardy-Weinberg equilibrium for all participants (Additional file 1: Table S1). Power analysis was performed using QUANTO 1.2 software. Power analysis showed that based on our sample size, we had 90.1% power for rs653765 and 64.3% power for rs514049 to detect a relative risk difference between genotypes at an odds ratio of 1.5 and a significance level of 0.05. Student’s *t*-test or the Mann-Whitney *U*-test was used for normally distributed and non-parametric data, respectively. Analysis of variance (ANOVA) was performed for all other calculations. All data are presented as the mean ± standard error of the mean (SEM). A difference was considered to be significant at a *P*-value <0.05.

## Results

### Clinical characteristics

The characteristics of the study participants are shown in Tables 1 and 2. In total, 890 participants were recruited in this study, 440 patients with sepsis and 450 healthy controls. No significant differences in age or gender distribution were detected between the cases and the controls (Table 1). The mean age was 61.31 ± 19.86 years for the patients with sepsis and 62.07 ± 13.45 years for the controls. Lung tissue (60.5%), abdominal tissue (16.4%) and trauma (12.5%) were the primary sources of infection. Gram-negative infection (32.0%), fungi (20.7%) and polymicrobial infection (16.6%) were the primary pathogens. *Acinetobacter baumannii* (25.9%, 7.5% were single bacterial strains), yeast sporophytes and Aspergillus (8.9%) were the primary pathogenic bacteria. The dysfunction of two or more organs was observed in 84.5% of the total patients. The percentages of patients with sepsis, severe sepsis and septic shock were 15.5%, 62.9% and 21.6%, respectively (data not shown). The 28-day mortality rate was 17.5% in this study cohort (Table 2).

**Table 2:** **Epidemiologic data of sepsis patients**

**Variable**	**Sepsis group (n = 440)**
Age, years, mean ± standard error of the mean	61.31 ± 19.86
Male/female, number	290/150
Primary diagnosis (%)	51 (11.6)
**Previous health status (%)**	
Previous surgery	55 (12.5)
Hypertension	61 (13.9)
Ischemic cardiac disease	36 (8.2)
**Source of injection**	
Lung	266 (60.5)
Blood	52 (11.8)
Abdomen	72 (16.4)
Urinary tract	16 (3.6)
Catheter-related	10 (2.3)
Trauma	55 (12.5)
Brain	29 (6.6)
Drainage liquid	7 (1.6)
Others	4 (0.9)
**Identified pathogen, n (%)**	
Gram-positive	46 (10.5)
Gram-negative	141 (32.0)
Mix of gram-positive and gram-negative	46 (10.5)
Fungus	91 (20.7)
Polymicrobial	73 (16.6)
Negative blood culture	46 (10.5)
**Pathogenic bacteria**	
*Acinetobacter baumannii*	114 (25.9)
Yeast sample sporphyte	39 (8.9)
Aspergillus	20 (4.5)
*Pseudomonas aeruginosa*	22 (5.0)
*Staphylococcus aureus*	20 (4.5)
*Staphylococcus haemolyticus*	16 (3.6)
*Escherichia coli*	13 (3.0)
Others	83 (18.9)
**Number of organs with dysfunction (%)**	
1	68 (15.5)
2	140 (31.8)
3 or more	232 (52.7)
APACHE II score, mean ± standard error of the mean	26.17 ± 6.13
28-day mortality, %	77 (17.5)

Results are presented as number (%) unless stated otherwise. APACHE, acute physiology and chronic health evaluation.

### Association of ADAM10 polymorphisms with sepsis susceptibility

The genotype and allele frequencies of the ADAM10 SNPs for the cases and the controls are presented in Tables 3 and 4. As shown in Table 3, no significant difference in any genotype or allele frequency was observed between the sepsis patients and the controls, suggesting that the ADAM10 SNPs may not affect the risk of sepsis. When dividing the cases into the subtypes of sepsis, severe sepsis and septic shock, our results revealed a significant difference in the genotype and allele frequencies of the rs653765 SNP between the sepsis and severe sepsis subtypes (*P* = 0.0014) or between the severe sepsis and septic shock subtypes (*P* = 0.0037) (Table 4). The C allele and the CC genotype of rs653765 displayed a trend toward promoting the development of sepsis. With regard to the other SNP, the AA genotype and the A allele of the rs514049 SNP displayed a significant difference between the septic shock and sepsis subtypes but not between the sepsis and severe sepsis subtypes (*P* = 0.428) or between the severe sepsis and septic shock subtypes (*P* = 0.210) (Table 4). Moreover, the frequencies of the haplotypes of the ADAM10 SNPs in the sepsis patients and the healthy controls were analyzed using Haploview software, but no significant differences were detected (Table 5).

**Table 3:** **Frequency distribution of rs653765 and rs514049 genotypes and alleles in sepsis patients and healthy controls**

	**Cases (n = 440) (%)**	**Controls (n = 450) (%)**	***P***	***P*** *	**Adjusted odds ratio (95% CI)**
**rs653765 C > T**					
CC	346 (78.6)	338 (75.1)	0.234	0.234	-
CT	82 (18.6)	91 (20.2)	-	-	-
TT	12 (2.7)	21 (4.7)	-	-	-
CC/CT	428 (97.3)	429 (95.3)	0.126	0.234	1.746 (0.848, 3.594)
TT/CT	94 (21.4)	112 (24.9)	0.213	0.234	0.820 (0.600, 1.121)
C	774 (88.0)	767 (85.2)	-	-	1.000 (reference)
T	106 (12.0)	133 (14.8)	0.091	0.234	0.790 (0.600, 1.039)
**rs514049 A > C**					
AA	388 (88.2)	394 (87.6)	0.954	1.000	-
AC	48 (10.9)	52 (11.6)	-	-	-
CC	4 (0.9)	4 (0.9)	-	-	-
AA/AC	436 (99.1)	446 (99.1)	1.000	1.000	0.978 (0.243, 3.935)
AC/CC	52 (11.8)	56 (12.4)	0.775	1.000	0.943 (0.630, 1.411)
A	824 (93.6)	840 (93.3)			1.000 (reference)
C	56 (6.4)	60 (6.7)	0.796	1.000	0.952 (0.653, 1.387)

*False discovery rate-adjusted *P*-value for multiple hypotheses testing using the Benjamin-Hochberg method.

**Table 4:** **Frequency distribution of rs653765 and rs514049 genotypes and alleles between sepsis subtypes**

	**Sepsis subtype**	**Severe sepsis**	**Septic shock**	**Severe sepsis/septic shock**	***P*** **1**	***P*** **2**	***P*** **3**	***P*** 1*	***P*** 2*	***P*** 3*
	**(n = 55) (%)**	**(n = 288) (%)**	**(n = 97) (%)**	**(n = 385) (%)**						
**rs653765 C > T**										
**CC**	35 (63.6)	238 (82.6)	73 (75.3)	311 (80.8)	0.0014	0.129	0.0037	0.0014	0.129	0.0037
**CT/TT**	20 (36.4)	50 (17.4)	24 (24.7)	74 (19.2)	-	-	-	-	-	-
**C**	87 (79.1)	520 (90.3)	167 (86.1)	687 (89.2)	0.0008	0.114	0.0023	0.0014	0.129	0.0037
**T**	23 (20.9)	56 (9.7)	27 (13.9)	83 (10.8)	-	-	-	-	-	-
**rs514049 A > C**										
**AA**	45 (81.8)	252 (87.5)	91 (93.8)	343 (89.1)	0.257	0.021	0.118	0.428	0.024	0.210
**AC/CC**	10 (18.2)	36 (12.5)	6 (6.2)	42 (10.9)	-	-	-	-	-	-
**A**	100 (90.9)	536 (93.1)	188 (96.9)	724 (94.0)	0.428	0.024	0.210	0.428	0.024	0.210
**C**	10 (9.1)	40 (6.9)	6 (3.1)	46 (6.0)	-	-	-	-	-	-

*P*1, sepsis group versus severe sepsis; *P*2, sepsis group versus septic shock; *P*3, sepsis group versus severe sepsis/septic shock. *False discovery rate-adjusted *P*-value for multiple hypotheses testing using the Benjamin-Hochberg method.

**Table 5:** **Frequencies of haplotypes of the** ***ADAM10*** **gene in sepsis patients and controls**

**rs514049-rs653765**	**Cases, n (%)**	**Controls, n (%)**	***P***	***P*** *	**Odds ratio (95% CI)**
**A-C**	786 (89.3)	781 (86.8)	-	-	1.000 (reference)
**A-T**	49 (5.6)	60 (6.7)	0.293	0.293	0.812 (0.549, 1.199)
**C-T**	45 (5.1)	59 (6.6)	0.174	0.293	0.758 (0.508, 1.131)

Values were adjusted for age and sex. *False discovery rate-adjusted *P*-value for multiple hypotheses testing using the Benjamin-Hochberg method.

### Influence of these ADAM10 polymorphisms on the expression levels of ADAM10

**Figure 1:**
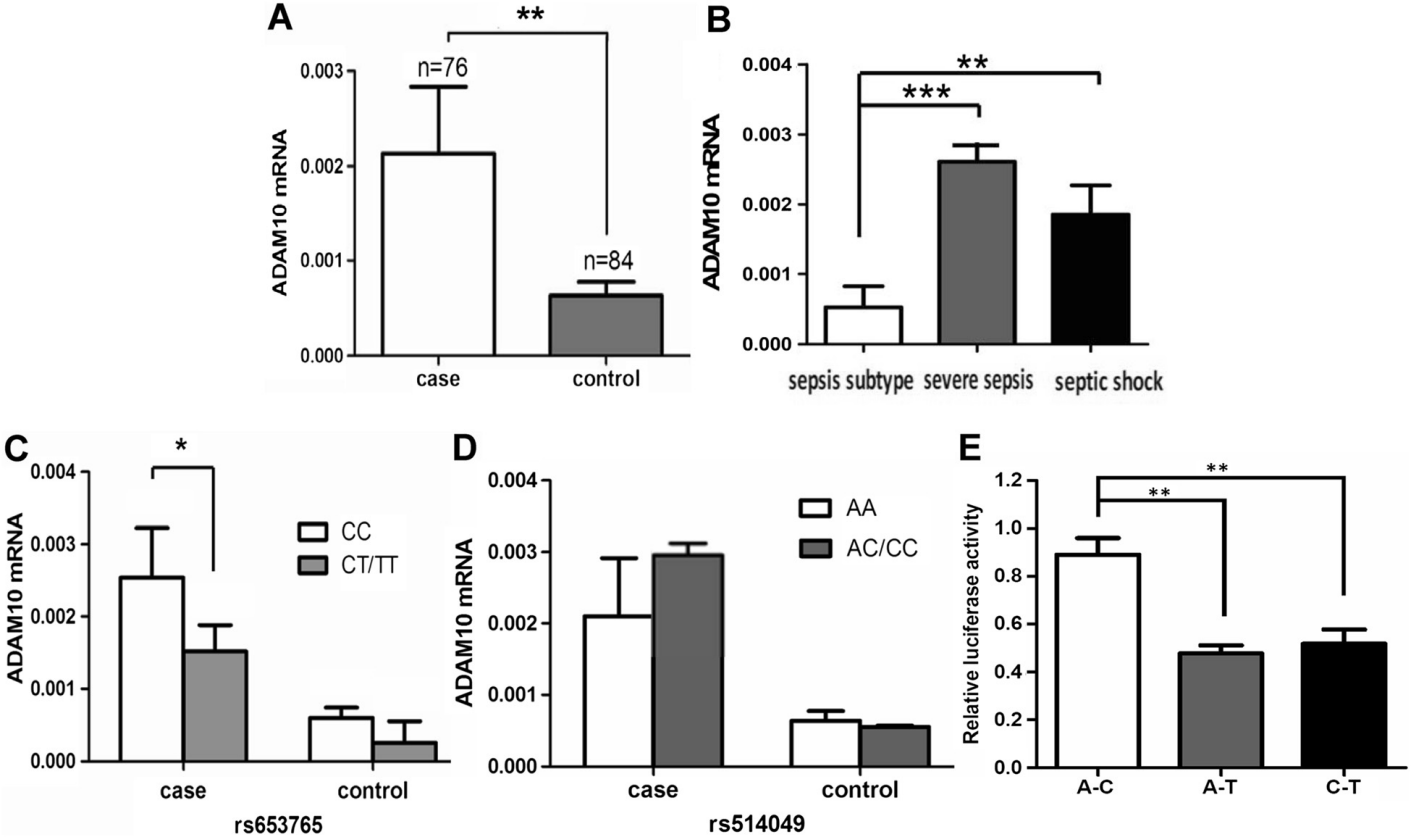
**Expression levels of A disintegrin and metalloproteinase 10 (ADAM10) polymorphisms in sepsis patients and healthy controls.** Expression levels of ADAM10 in sepsis patients and healthy controls **(A)**. Expression levels of ADAM10 in sepsis, severe sepsis and septic shock subgroup **(B)**; the genotype distribution between the rs653765 polymorphisms and the expression of the ADAM10 gene **(C)**; the genotype distribution between the rs514049 polymorphisms and the expression of the ADAM10 gene in severe sepsis **(D)**; effect of rs514049-rs653765 haplotypes on transcriptional activity of ADAM10. In luciferase assays in THP-1 cells, luciferase activity was presented as a ratio (Firefly/Renilla) **(E)**; the horizontal line represents the mean expression level of ADAM10 with each group. *P <0.05; **P <0.01; ***P <0.001.

The difference in the ADAM10 mRNA expression level between each genotype was further analyzed in the sepsis patients. As shown in Figure 1A, we found that the sepsis patients displayed a significantly higher ADAM10 mRNA expression level than the healthy controls (Figure 1A, *P* <0.01). When the sepsis patients were divided into subgroups, we observed that ADAM10 expression was significantly higher in the patients with severe sepsis or septic shock than in the patients with sepsis (Figure 1B, both *P* <0.01). Next, we analyzed the genotype distribution of ADAM10 mRNA expression to investigate the possible relationship between these polymorphisms and the expression of the ADAM10 gene in severe sepsis patients. As shown in Figure 1C, the rs653765 CC carriers exhibited significantly higher ADAM10 mRNA expression levels than the patients who carried the CT or TT genotype (*P* <0.05), whereas the healthy patients who carried the same polymorphic alleles displayed no significant difference (Figure 1C). For the rs514049 polymorphism, no significant differences in the ADAM10 expression levels were observed in the genotypes between the cases or the controls (Figure 1D). Furthermore, we performed luciferase assays using the THP-1 cell line to characterize the rs514049-rs653765 ADAM10 promoter haplotypes and found that the ADAM10 C-A haplotype carriers displayed significantly higher reporter activity than the T-A carriers and the T-C carriers (Figure 1E), suggesting that the C allele of rs653765 may the primary factor that increases ADAM10 expression in THP-1 cells. These results are consistent with our previous case-control results, suggesting that rs653765 is a functional SNP that may be involved in the pathology of sepsis via the regulation of ADAM10 mRNA expression.

### Association between these ADAM10 polymorphisms and the expression levels of related cytokines

**Figure 2:**
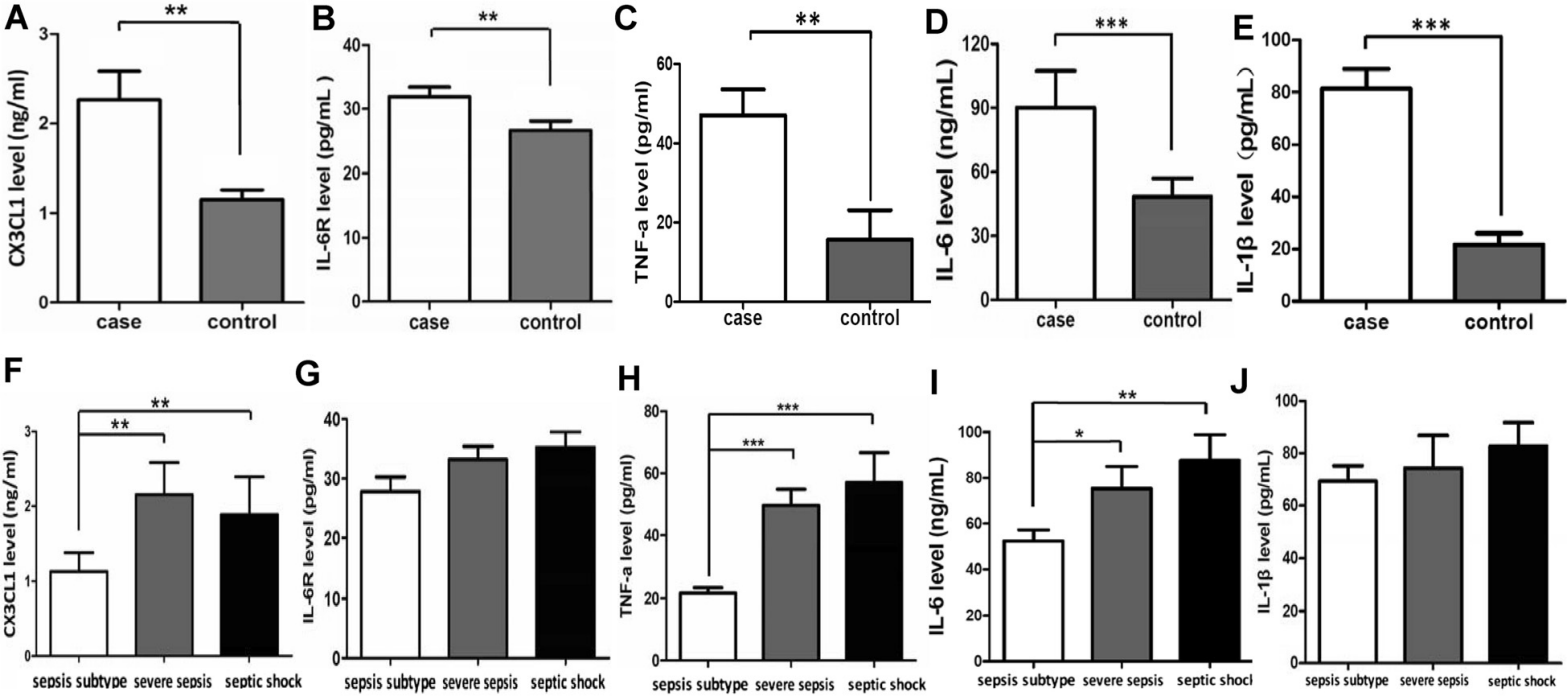
**Expression levels of CX3CL1 (A), IL-6R (B), TNF-** **α** **(C), IL-6 (D) and IL-1** **β** **(E) between the sepsis patients and healthy controls, and expression levels of CX3CL1 (F), IL-6R (G), TNF-** **α** **(H), IL-6(I) and IL-1** **β** **(J) in the sepsis, severe sepsis and septic shock subgroups.** The horizontal line represents the mean expression level of ADAM10 with each group. *P <0.05; **P <0.01; ***P <0.001.

**Figure 3:**
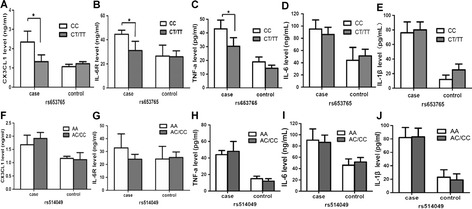
**The genotype distribution between the rs653765 polymorphisms and the expression of CX3CL1 (A), IL-6R (B), TNF-** **α** **(C), IL-6 (D) and IL-1** **β** **(E) in severe sepsis patients and controls, and genotype distribution between the rs514049 polymorphisms and the expression of CX3CL1 (F), IL-6R (G), TNF-** **α** **(H), IL-6(I) and IL-1** **β** **(J) in severe sepsis patients and controls.** The horizontal line represents the mean expression level of ADAM10 with each group. *P <0.05; **P <0.01; ***P <0.001.

The differences in the expression levels of ADAM10 substrates (CX3CL1, IL-6R and TNF-a) and the pro-inflammatory cytokines IL-1ß and IL-6 according to the ADAM10 genotype were analyzed in PBMCs from the sepsis patients and the controls. Our results indicated that the expression of CX3CL1, IL-6R, TNF-a, IL-1ß and IL-6 were significantly higher in the sepsis patients (subtype) than in the healthy controls (Figure 2A-D). Among the three patient subgroups, the severe sepsis patients displayed the highest CX3CL1 expression level (Figure 2F), whereas the sepsis subtype patients displayed the lowest expression of TNF-a, IL-6 and IL-1β (Figure 2H-J). However, no significant differences in IL-6R expression were observed between the subgroups (Figure 2G). Next, we analyzed the differences in these cytokines according to the ADAM10 genotype. Our results revealed that the ADAM10 rs653765 CC genotype carriers exhibited a significant increase in the expression of the three substrates of ADAM10 (CX3CL1, IL-6R and TNF-a) compared with the rs653765 CT/TT carriers in the subgroup of severe sepsis patients (Figure 3A-C) but that the expression levels of the pro-inflammatory cytokines IL-1β and IL-6 were not significantly associated with the rs653765 polymorphism (Figure 3D,E). In addition, no significant differences were observed between the rs514049 polymorphisms and the sepsis-related substrates that we measured in our study (Figure 3F-J).

## Discussion

Increasing evidence has demonstrated that ADAM10 is intimately linked to inflammation and may mediate inflammation-related biological and pathological processes [33-35]. A recent study reported that ADAM10 induces the loss of endothelial barrier function in septic mice and that the severity of cellular injury and sepsis is mitigated by ADAM10 inhibition [22], which led us to investigate the relationship between ADAM10 and sepsis. To the best of our knowledge, our study is the first to investigate the association between two functional polymorphisms (rs653765 and rs514049) in the ADAM10 gene and the risk of sepsis. No statistically significant differences between sepsis cases and controls in the genotype or allele frequencies were observed, suggesting that the ADAM10 SNPs may not be the risk factor for the occurrence of sepsis. The novel findings from a secondary analysis were that the rs653765 C allele and CC genotype of rs653765 displayed a trend toward promoting the development of sepsis from the sepsis to severe sepsis subtype, and the CC genotype carriers was associated with higher ADAM10 mRNA expression in the severe sepsis subtype than in the sepsis (subtype) and further influenced the expression of ADAM10 substrates in the severe sepsis subtype, thereby potentially contributing to the progression from sepsis to severe sepsis.

Several lines of evidence have confirmed that these ADAM10 SNPs affect mRNA expression and are involved in various inflammation-related diseases [28,29]. A recent study has shown that nucleotides −2179 to −1 of the ADAM10 gene represent functional TATA-less promoters [24]. Bekris *et al*. discovered that the ADAM10 rs514049-rs653765 C-A promoter haplotype, especially the rs514049 C allele, is associated with a higher cerebral spinal fluid sAPPa level in cognitively normal controls than in patients with AD [28]. Another previous study showed that the rs653765 C > T polymorphism is located downstream of the potential binding site for the myc-associated zinc finger protein transcription factor, suggesting that the rs653765 polymorphism may be a functional SNP [24]. In addition, Li *et al*. demonstrated a positive association between the ADAM10 polymorphism rs653765 and ACI risk [29]. Consequently, in the present study, we investigated the association of these two functional SNPs, rs514049 and rs653765, which are located in the promoter region of the ADAM10 gene, with the risk of sepsis. Our initial results did not reveal a significant difference between the sepsis patients and the healthy controls, suggesting that these SNPs may not be related to the occurrence of sepsis. However, when we divided the sepsis sample into subgroups according to the severity of sepsis, we found that the C allele and the CC genotype of the rs653765 SNP resulted in a higher risk for the severe sepsis subtype than for the sepsis subtype. Moreover, among the patients with severe sepsis, the rs653765 CC genotype carriers exhibited a higher ADAM10 expression level than the rs653765 CT/TT carriers, suggesting than this SNP may be functional. To further confirm this result, we perform a luciferase activity assay using the THP-1 cell line consisting of three rs514049 – rs653765 ADAM10 promoter haplotypes to determine which haplotype exerts an effect on the expression of ADAM10 expression. The C-A variants displayed a higher level of luciferase activity than the T-A and T-C variants in the THP-1 cells, suggesting that rs653765 may be a functional SNP and that the C allele may increase the expression of ADAM10. Furthermore, we found that the ADAM10 expression level in the sepsis subgroups was consistent with the results of our case-control study and that the healthy controls did not display altered ADAM10 expression or the differential expression of ADAM10 between the different genotypes. These results indicated that ADAM10 may influence the progression of sepsis rather than the occurrence of sepsis. However, another SNP of the ADAM10 gene, the rs514049 A > C polymorphism, which has been reported to perform a similar function to the rs653765 SNP, was not significantly associated with the expression of ADAM10 in our study. We speculate that this result could be attributed to the binding of different promoters to distinct regions of these SNPs. Our subsequent study will focus on the molecular mechanisms underlying these two functional SNPs utilizing a promoter prediction method and the experimental confirmation of our results in a cellular model of sepsis.

We further detected the downstream substrates of ADAM10 in PBMCs and attempted to determine whether the ADAM10 polymorphism further affects the expression levels of its substrates. CX3CL1 induces the detachment of monocytes and guides leukocytes to the site of inflammation, and ADAM10 is the major protease involved in the release of CX3CL1 under pro-inflammatory conditions [36-38]. IL-6R is primarily generated via ADAM10-mediated ectodomain shedding, and inducing IL-6 trans-signaling leads to inflammatory processes [39-41]. In addition to its role as a pro-inflammatory cytokine, recent studies have indicated that ADAM10 may also function as a sheddase of TNF-α in cells [41,42]. Therefore, three representative ADAM10 substrates, CX3CL1, IL-6R and TNF-a, were selected for analysis in this study. We found that the severe sepsis patients expressed significantly higher levels of CX3CL1, IL-6R, and TNF-a than the healthy controls. Importantly, we found that among the subgroup of patients with severe sepsis, the rs653765 CC genotype carriers expressed significantly higher levels of CX3CL1, IL-6R and TNF-a than the CT/TT carriers. This finding is consistent with our previous results describing the association between the rs653765 polymorphism and the risk of severe sepsis and supports the hypothesis that the ADAM10 rs653765 CC genotype contributes to the pathogenesis of severe sepsis by increasing the ADAM10 mRNA expression level, accompanied by the upregulation of its substrates.

Evidence indicates that ADAM10 is involved in the development and progression of inflammation via various cellular processes. For instance, ADAM10 induces the secretion of IL-1β and IL-6 in macrophages by activating the extracellular signal-regulated kinase 1/2 and NF-κB signaling pathways [43]. In addition, ADAM10-Dll4 signaling represents a major signaling pathway in human endothelial cells that drives the inflammatory events involved in the release of IL-6 [44]. Furthermore, both the ADAM10 inhibitor GI254023X and ADAM10 siRNA block the inflammatory process by decreasing vascular permeability and the expression levels of pro-inflammatory cytokines [22,23]. To determine whether this functional ADAM10 SNP ultimately influences the expression of pro-inflammatory cytokines, we investigated the expression levels of IL-1ß and IL-6 in PBMCs from patients with severe sepsis. We found that the patients with sepsis expressed significantly higher IL-1ß and IL-6 levels than the healthy controls, as predicted. Nevertheless, no significant associations were observed between the ADAM10 polymorphisms and these pro-inflammatory cytokines in the severe sepsis patients. We speculate that several reasons may contribute to this result. Because the inflammatory system is vast and complex, the increase in the ADAM10 mRNA level attributable to the rs653765 CC genotype may not be sufficiently strong to significantly influence inflammatory signaling during the development of severe sepsis. Alternatively, ADAM10 may ultimately influence the risk of severe sepsis via other inflammation- or non-inflammation-related pathways aside from those which we examined. In contrast, it is difficult to achieve strict homogeneity under the ever-changing pathogenic conditions of the sepsis patients in this study, which may have contributed to this negative result. Nevertheless, further investigation is required to confirm these results.

Furthermore, the allele frequencies of the ADAM10 polymorphisms differ in distinct healthy populations. In the Chinese Han population examined in our study, the rs653765 allele frequency was 85.2% for the C allele, and the rs514049 allele frequency was 93.3% for the A allele; these allele frequencies are similar to previous data in Chinese individuals (86.5% for the rs653765 C allele and 94.3% for the rs514049 A allele) [29], whereas another study of white individuals showed that the rs653765 C allele frequency was 24.1% and that the rs514049 A allele frequency was 39.8% [45], which are far lower than the frequencies observed in the Han Chinese populations. Ethnic differences may contribute to this diversity. Notably, the associations between the two functional SNPs of ADAM10 and disease have been reported in only three articles to date [28,29,45]. Further studies of these polymorphisms using larger sample sizes and in different ethnicities should be conducted.

Some potential limitations in our study should be addressed. For now no study about the association between these ADAM10 SNPs and sepsis has been reported in other population, and we still need the validation cohort study for further confirm our results, further investigation with a larger and more ethnically diverse population of septic patients is warranted to support our preliminary conclusions. Moreover, although the recruited septic patients who have certain diseases were excluded in our study for the homogeneity of samples, there is also a possibility that ADAM10 genotype were inversely associated with diseases that were excluded.

## Conclusion

In the present study, for the first time, we demonstrated that individuals carrying the functional variant rs653765 in the promoter region of ADAM10 may exhibit susceptibility to the development of sepsis. Rs653765 SNP is a functional SNP in severe sepsis, and the CC genotype may increase the ADAM10 mRNA expression level, thereby influencing the levels of ADAM10 substrates, which may ultimately contribute to the risk for the development of sepsis. However, we did not observe any association between the ADAM10 polymorphisms and the levels of pro-inflammatory cytokines in the severe sepsis patients. Future studies will focus on the mechanisms by which this functional SNP (rs653765) is involved in the pathogenesis of sepsis.

## Key messages

- For the first time exploring the relationship between ADAM10 polymorphisms and sepsis.
- Individuals with a functional variant (rs653765) in the promoter region of ADAM10 may have susceptibility to the development of sepsis
- The risk CC genotype of rs653765 could functionally affect the expression level of ADAM10 mRNA and accompanied with the upregulation of the expression levels of its substrates in severe sepsis
- This risk polymorphism may confer the susceptibility to the development of sepsis more than through inflammatory pathways

## Additional file

Additional file 1: Table S1. Hardy-Weinberg equilibrium assay for rs653765 and rs514049 genotypes in sepsis patients and healthy controls.

## Abbreviations

ACI: atherosclerotic cerebral infarction
AD: Alzheimer’s disease
ADAM10: A disintegrin and metalloproteinase 10
ANOVA: analysis of variance
APACHE II: acute physiology and chronic health evaluation II
cDNA: complementary deoxyribonucleic acid
CX3CL1: Fractalkine
ELISA: enzyme-linked immunosorbent assay
IL-1β: interleukin-1 beta
IL-6: interleukin-6
IL-6R: interleukin-6 receptor
PBMC: peripheral blood mononuclear cell
QUANTO: quantity adjusting option
RT-PCR: real-time quantitative polymerase chain reaction
siRNA: small interfering ribose nucleic acid
SNP: single-nucleotide polymorphism
THP-1: human acute monocytic leukemia cell line
TNF-a: tumor necrosis factor alpha
