# Supplementary material for: An ADAM10 promoter polymorphism is a functional variant in severe sepsis patients and confers susceptibility to the development of sepsis
Source: Crit Care. 2015 Mar 5;19(1):73. doi: 10.1186/s13054-015-0796-x (PMC4373036; doi:10.1186/s13054-015-0796-x)
Supplement: Additional file 1: Table S1. — Hardy-Weinberg equilibrium assay for rs653765 and rs514049 genotypes in sepsis patients and healthy controls. [file 13054_2015_796_MOESM1_ESM.doc]

Supplementary Table 1. The Hardy-Weinberg equilibrium assay for rs653765 and rs514049 genotypes in sepsis patients and healthy controls

| **Zhanjiang (Southern China)** |  |  |  | P-value |
| --- | --- | --- | --- | --- |
| rs653765 C>T |  |  |  |  |
| Case (n=273) | CC(216) | CT(50) | TT(7) | 0.057 |
| Control(n=280) | CC(213) | CT(58) | TT(9) | 0.050 |
| rs514049 A>C |  |  |  |  |
| Case (n=273) | AA(240) | AC(31) | CC(2) | 0.375 |
| Control(n=280) | AA(246) | AC(32) | CC(2) | 0.402 |
| **Harbin (Northern China)** |  |  |  |  |
| rs653765 C>T |  |  |  |  |
| Case (n=167) | CC(130) | CT(32) | TT(5) | 0.096 |
| Control(n=170) | CC(125) | CT(33) | TT(12) | 0.054 |
| rs514049 A>C |  |  |  |  |
| Case (n=167) | AA(148) | AC(17) | CC(2) | 0.078 |
| Control(n=170) | AA(148) | AC(20) | CC(2) | 0.177 |
| **ALL** |  |  |  |  |
| rs653765 C>T |  |  |  |  |
| Case (n=440) | CC(346) | CT(82) | TT(12) | 0.012 |
| Control(n=450) | CC(338) | CT(91) | TT(21) | 0.111 |
| rs514049 A>C |  |  |  |  |
| Case (n=440) | AA(388) | AC(48) | CC(4) | 0.076 |
| Control(n=450) | AA(394) | AC(52) | CC(4) | 0.130 |
